# Increased breast cancer mortality due to treatment delay and needle biopsy type: a retrospective analysis of SEER-medicare

**DOI:** 10.1007/s12282-023-01456-3

**Published:** 2023-05-03

**Authors:** Rashmi Pathak, Macall Leslie, Priya Dondapati, Rachel Davis, Kenichi Tanaka, Elizabeth Jett, Inna Chervoneva, Takemi Tanaka

**Affiliations:** 1 Stephenson Cancer Center, University of Oklahoma Health Sciences Center, 975 NE 10th, BRC-W, Rm 1415, Oklahoma City, OK 73104 USA; 2 Department of Surgery, University of Oklahoma Health Sciences Center, 975 NE 10th, Oklahoma City, OK 73104 USA; 3 Department of Anesthesiology, University of Oklahoma Health Sciences Center, 920 SL Young Blvd, WP1140, Oklahoma City, OK 73104 USA; 4 Department of Radiology, University of Oklahoma Health Sciences Center, 800 SL Young Blvd, Oklahoma City, OK 73104 USA; 5 Department of Pharmacology, Physiology and Cancer Biology, Division of Biostatistics, Thomas Jefferson University, 130 S. 9th Street, 17th Floor, Philadelphia, PA 19107 USA; 6 Department of Pathology, University of Oklahoma Health Sciences Center, 975 NE 10th, Oklahoma City, OK 73104 USA

**Keywords:** Core needle biopsy, Vacuum-assisted biopsy, Breast cancer-specific mortality, Treatment delay

## Abstract

**Background:**

Substantial evidence indicates that delay of first treatment after diagnosis is associated with poorer survival outcomes in breast cancer. Accordingly, the Commission on Cancer introduced a quality measure for receipt of therapeutic surgery within 60 days of diagnostic biopsy for stage I–III breast cancer patients in the non-neoadjuvant setting. It is unknown, however, what may contribute to mortality associated with treatment delay. Therefore, we investigated whether biopsy type moderates the effect of the mortality risk posed by treatment delay.

**Methods:**

Retrospective analysis of 31,306 women with stage I–III breast cancer diagnosed between 2003 and 2013 selected from the SEER-Medicare database was performed to determine whether needle biopsy type [core needle biopsy (CNB) or vacuum-assisted biopsy (VAB)] impacts time to treatment (TTT)-associated survival outcomes. Multivariable Fine-Gray competing risk survival models, adjusted for inverse propensity score weights, were used to determine the association between biopsy type, TTT, and breast cancer-specific mortality (BCSM).

**Results:**

TTT ≥ 60 days was associated with 45% higher risk of BCSM (sHR = 1.45, 95% CI 1.24–1.69) compared to those with TTT < 60 days in stage I–III cases. Independent of TTT, CNB was associated with 28% higher risk of BCSM compared to VAB in stage II–III cases (sHR = 1.28, 95% CI 1.11–1.36), translating to a 2.7% and 4.0% absolute difference in BCSM at 5 and 10 years, respectively. However, in stage I cases, the BCSM risk was not associated with type of biopsy.

**Conclusions:**

Our results suggest that treatment delay ≥ 60 days is independently associated with poorer survival outcomes in breast cancer patients. In stage II–III, CNB is associated with higher BCSM than VAB. However, type of biopsy does not underlie TTT-associated breast cancer mortality risk.

**Supplementary Information:**

The online version contains supplementary material available at 10.1007/s12282-023-01456-3.

## Introduction

Breast cancer remains a significant cause of morbidity and mortality among women worldwide. Advances in care over the past decades have offered improved diagnostic clarity along with expanded treatment options—greatly improving outcomes as well as the quality of life for women with breast cancer [1–5]—yet have also, in part, caused increasing treatment delays [6–10]. Substantial evidence supports the association between treatment delays and higher breast cancer mortality [11–17]. We have previously reported that delay of surgery over 60 days after biopsy is positively associated with disease progression in T1N0M0 hormone receptor-positive breast cancer patients [18]. Correspondingly, the Commission on Cancer introduced a new quality measure in 2022, recommending surgery within 60 days of diagnostic biopsy for stage I–III breast cancer patients in the non-neoadjuvant setting [19]. However, it remains unknown what factors may underlie the breast cancer mortality risk associated with treatment delay.

Breast malignancies are definitively diagnosed by pathological evaluation of suspicious lesions collected by various biopsy methods (e.g., fine-needle aspiration, core needle, vacuum-assisted, or open surgical excision biopsy) [20]. Migration of the preferred sampling method from open surgical excision biopsy immediately followed by the same-day surgical resection to needle biopsy followed by subsequent consultation and treatment has undoubtedly created an interval between diagnosis and treatment initiation that may be prolonged. The type of biopsy used for sampling is typically chosen based on clinical characteristics of suspicious lesion(s) (e.g., size, location, and shape) as well as the preference of clinicians or facilities [20, 21]. Currently, core needle biopsies (CNB) and vacuum-assisted biopsies (VAB) are the most commonly employed methods for sampling breast lesions to allow cytomorphological evaluation and molecular/biological subtyping [22]. A recent retrospective analysis of matched surgical breast cancer cases with tumor size ≤ 30 mm, treated with or without postoperative radiation therapy, showed earlier onset and higher rates of distant metastasis at 5–10 years after diagnosis among those diagnosed by CNB (*n* = 1729) compared to fine-needle aspiration biopsy (FNA, *n* = 354) [23]. Additionally, one prospective study indicated approximately 13–15% higher incidence of sentinel node metastases in invasive breast cancer patients diagnosed by either CNB or FNA, respectively, compared to those who received excisional biopsy [24]. While these studies represent potential unintended negative impacts of needle biopsy on breast cancer disease progression and survival, no study has investigated a causal relationship between biopsy type and treatment delay on breast cancer mortality. Therefore, we used the SEER-Medicare database to explore whether the risk of breast cancer-specific mortality (BCSM) due to treatment delay is attributable to the type of biopsy.

## Materials and methods

### Cohort

**Fig. 1:**
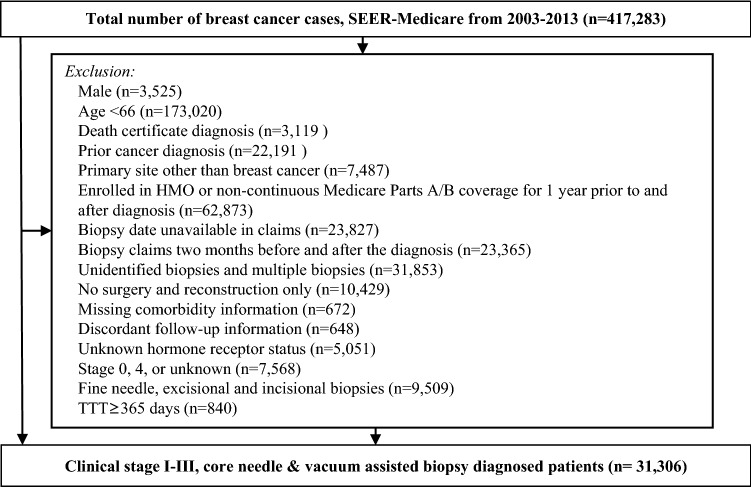
Exclusion scheme

Women diagnosed by core needle (CNB) or vacuum-assisted biopsy (VAB) with stage I–III breast cancer between 2003 and 2013 were selected from the Surveillance, Epidemiology, and End-Results (SEER)-Medicare linked database. The SEER-Medicare linked database combines Medicare Parts A and B claims with clinical and outcome data from SEER cancer registries from 12 main geographic areas (Connecticut, Detroit, Hawaii, Iowa, New Mexico, Seattle/Puget Sound, Utah, Kentucky, Louisiana, New Jersey, Georgia, and California). Only patients who had continuous Medicare Parts A and B enrollment for at least 1 year prior through 1 year after diagnosis and were not enrolled in a Medicare Advantage (i.e., health maintenance organization) plan during any part of that period were selected to ensure accurate capture of patient claims (Fig. 1). Eighty-seven percent cases in the original dataset had at least one identified needle biopsy claim during a ± 1-month window from the month and year of diagnosis identified in the SEER Patient Entitlement and Diagnosis Summary File, and approximately 7.7% of those cases had multiple types or dates of needle biopsy and were excluded from our analysis. Patients who initiated treatment in fewer than 8 or over 365 days after diagnosis were excluded.

### Exposure(s)

Two primary exposures were examined in this study: (1) time to treatment (TTT), defined as the number of days between the diagnostic biopsy and initiation of treatment (surgery or neoadjuvant therapy), and (2) type of needle biopsy used for diagnosis (CNB vs. VAB). TTT was assessed using a binary indicator of < 60 or ≥ 60 days.

### Outcome

Breast cancer-specific mortality (BCSM) in the presence of competing events (i.e., death from other causes) was examined as the primary outcome. Survival times were calculated in months as the time from diagnosis (i.e., biopsy) to death (*event*) or loss to follow-up (*censored*).

### Definitions

Breast biopsy procedures and treatment claims were identified by relevant Healthcare Common Procedure Coding System (HCPCS) and International Classification of Disease, 9th Revision (ICD-9) diagnostic and procedure codes (Supplementary Data 1). SEER registry data were used to identify patient demographic and clinical characteristics. Age at the time of diagnosis (i.e., first biopsy) was categorized by 5-year intervals (≤ 69, 70–74, 75–79, and ≥ 80). Patient race was defined as Black, White, or other (American Indian, Hawaiian, Asian, and other racial groups). Group stage was determined using the AJCC 6th edition and classified as I, II, or III. Tumor histology was classified using the ICD-O-3 system as ductal, lobular, and other (Supplementary Data 2). Hormone receptor status (estrogen/progesterone) was classified as positive or negative. HER2 status was defined as positive, negative, borderline, and unknown. Furthermore, surgery type was defined based on HCPCS and ICD-9 procedure codes and categorized as breast-conserving surgery, mastectomy, or mastectomy with immediate reconstruction. Similarly, receipt of chemotherapy was classified using HCPCS and ICD-9 codes (Supplementary Data 1).

### Statistical methods

The association between TTT and biopsy type, clinical characteristics, demographics, and treatments was evaluated using the Chi-square test. The effects of TTT and type of biopsy on BCSM were further analyzed using Fine-Gray competing risk models. To account for potential bias or imbalance in covariates, all the models were adjusted using the inverse propensity score weights (IPW) [25]. IPW was based on the propensity model for biopsy type as a response and socio-demographic and clinical factors predictors, including age, race, stage, grade, histology, hormonal receptor status, and HER2 status. Covariate balancing propensity scores were estimated using the R package “CBPS” [26], and balance was assessed using Love plots (Supplementary Data 3). Final Fine-Gray competing risk survival models were adjusted by normalized IPW, and included biopsy type, time to treatment, and stage as predictors of BCSM. Due to both direct and indirect effects of the stage with BCSM and biopsy type, respectively, it was assessed in both IPW and direct adjustment. Furthermore, the interactions between TTT, biopsy type, and stage were evaluated, but only significant interaction between biopsy type and the stage was retained in the final model. Subdistribution hazard ratios (sHR) were calculated with corresponding 95% confidence intervals. The final adjusted Fine-Gray models were used to derive the cumulative incidence function (CIF) based on a given set of baseline covariates with specifications for both binary indicators of biopsy type, TTT, and final interaction terms. Primary statistical analyses were conducted using SAS (version 9.4; Cary, NC), IPW using R [27], and graphs were generated using JMP Pro 15.2.0 (SAS; Cary, NC).

## Results

### Characterization of the cohort

Following exclusions, the final cohort included 31,306 women with stage I–III invasive breast cancer who underwent CNB or VAB between 2003 and 2013 from the SEER-Medicare database (Fig. 1). The median age at diagnosis was 75 (Q1–Q3: 70–80, range 66–105), and the median follow-up time was 3.8 years (Q1–Q3: 1.7–6.7 years; range: 0 months to 10.9 years). The median TTT was 27 days (Q1–Q3; 18–41 days) and was similar between patients who received surgery first (*n* = 30,344; mean: 25 days, Q1–Q3: 17–36 days) or neoadjuvant chemotherapy (*n* = 962; mean: 27 days, Q1–Q3: 20–40 days). Approximately 92.5% (*n* = 28,961) of patients had TTT < 60 days, and 7.5% (*n* = 2,345) had ≥ 60 days. A greater proportion of cases with TTT ≥ 60 days were over 80 years old (38.1% vs. 28.2%; *P* < 0.001) or Black (12.2% vs. 6.4%; *P* < 0.001; Table 1) compared to patients with TTT < 60 days. Cases with TTT ≥ 60 days were more likely to have stage II or III disease than those with TTT < 60 days (51.9% vs. 42.3%; *P* < 0.001) and less likely to receive breast-conserving surgery (57.1% vs. 63.8%; *P* = 0.002).

**Table 1:** Distribution of demographic and clinical characteristics by TTT

	TTT^a^ < 60 days *n* (%)	TTT ≥ 60 days *n* (%)	*P* value^b^
	28,961 (100)	2345 (100)	
Biopsy types			
VAB	11,615 (40.1)	929 (39.6)	0.64
CNB	17,346 (59.9)	1416 (60.4)	
Age			
69	6557 (22.6)	443 (18.9)	< 0.001
70–74	7623 (26.3)	521 (22.2)	
75–79	6613 (22.8)	487 (20.8)	
> 80	8168 (28.2)	894 (38.1)	
Race			
White	25,361 (87.6)	1879 (80.1)	< 0.001
Black	1846 (6.4)	286 (12.2)	
Other	1754 (6.1)	180 (7.7)	
Grade			
1–2	21,415 (73.9)	1752 (74.7)	0.41
3	7546 (26.1)	593 (25.3)	
Stage^c^			
I	16,710 (57.7)	1127 (48.1)	< 0.001
II–III	12,251 (42.3)	1218 (51.9)	
Histology			
Ductal	22,161 (76.5)	1722 (73.4)	0.002
Lobular	4839 (16.7)	452 (19.3)	
Other	1961 (6.8)	171 (7.3)	
Hormone receptor			
Positive	24,986 (86.3)	2030 (86.57)	0.692
Negative	3975 (13.7)	315 (13.4)	
HER2			
Positive	1190 (4.1)	102 (4.4)	< 0.001
Negative	11,063 (38.2)	1042 (44.4)	
Borderline	298 (1.0)	26 (1.1)	
Unknown	16,710 (56.7)	1175 (50.1)	
Type of Surgery			
Breast-conserving surgery	18,479 (63.8)	1340 (57.1)	< 0.001
Mastectomy	9747 (33.7)	890 (38.0)	
Mastectomy with reconstruction	735 (2.5)	115 (4.9)	
Treatment sequence			
Neoadjuvant	865 (3.0)	97 (4.1)	0.002
Surgery first	28,096 (97.0)	2248 (95.9)	

^a^Time to treatment is defined as time from diagnosis of breast cancer to definitive treatment (surgery or neoadjuvant therapy)

^b^*P *value is derived from the Chi-square test of independence

^c^Group stage derived from AJCC 6th or 7th ed. Staging Manual

Considerable differences in the type of biopsy received were noted by age and stage, with cases over 80 years old (30.7% vs. 26.4%; *P* < 0.001) or with stage II–III cancer (45.5% vs. 39.3%; *P* < 0.001) more likely to have received CNB compared to VAB (Table 2). While a smaller proportion of patients were diagnosed by VAB (*n* = 12,544, 40.1%) compared to CNB (*n* = 18,762, 59.9%) overall, there was no difference in TTT in either group (7.4% of VAB vs. 7.6% of CNB with TTT ≥ 60 days; *P* = 0.642).

**Table 2:** Distribution of demographic and clinical characteristics by biopsy type

	Vacuum-assisted biopsy	Core needle biopsy	*P* value^b^
	*n* (%)	*n* (%)	
TTT^a^			
< 60 days	11,615 (92.6)	17,346 (92.5)	0.642
≥ 60 days	929 (7.4)	1416 (7.6)	
Age			
< 69	3018 (24.1)	3982 (21.2)	< 0.001
70–75	3375 (26.9)	4769 (25.4)	
75–80	2843 (22.7)	4257 (22.7)	
> 80	3308 (26.4)	5754 (30.7)	
Race			
White	11,158 (89.0)	16,082 (85.7)	< 0.001
Black	772 (6.2)	1360 (7.3)	
Others	614 (4.8)	1320 (7.0)	
Grade			
1–2	9407 (75.0)	13,760 (73.3)	0.001
3	3137 (25.0)	5002 (26.7)	
Stage^c^			
I	7617 (60.7)	10,220 (54.5)	< 0.001
II–III	4927 (39.3)	8542 (45.5)	
Histology			
Ductal	9616 (76.7)	14,267 (76.0)	0.263
Lobular	2107 (16.8)	3184 (17.0)	
Other	821 (6.5)	1311 (7.0)	
Hormone receptor			
Positive	10,916 (87.0)	16,100 (85.8)	0.002
Negative	1628 (13.0)	2662 (14.2)	
HER2			
Positive	570 (4.5)	722 (3.9)	< 0.001
Negative	5702 (45.5)	6403 (34.1)	
Borderline	131 (1.0)	193 (1.0)	
Unknown	6141 (49.0)	11,444 (61.0)	
Type of surgery			
Breast conserving	8170 (65.1)	11,649 (62.1)	< 0.001
Mastectomy	3983 (31.8)	6654 (35.5)	
Mastectomy with reconstruction	391 (3.1)	459 (2.5)	
Treatment sequence			
Neoadjuvant	356 (2.8)	572 (3.0)	0.281
Surgery first	12,188 (97.2)	18,190 (97.0)	

^a^Time to Treatment is defined as time from diagnosis of breast cancer to definitive treatment (surgery or neoadjuvant therapy)

^b^*P* value is derived from the Chi-square test of independence

^c^Group stage derived from AJCC 6th or 7th ed. Staging Manual

### TTT is associated with an increased BCSM

**Fig. 2:**
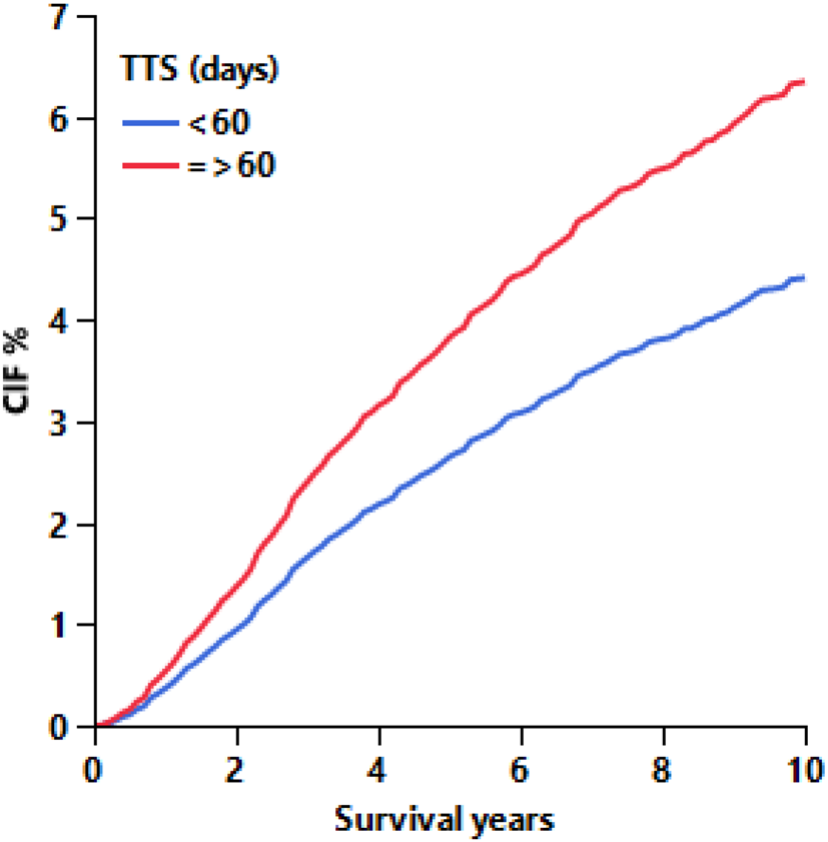
Adjusted cumulative incidence function of breast cancer-specific mortality by TTT

Adjusted Fine-Gray analysis of the main effects found that TTT ≥ 60 days was significantly associated with an increased risk of BSCM. Cases with TTT ≥ 60 days had 45% higher BCSM risk compared to TTT < 60 days (sHR: 1.45; 95% CI 1.24–1.69;  *P* < 0.001; Table 3), translating to approximately 1.2% greater (3.8% vs. 2.6%) 5-year and 1.9% greater (6.3% vs. 4.4%) 10-year cumulative incidence of BCSM (Fig. 2). Supplementary Data 4 shows the results of the propensity model for biopsy type used for calculating the IPWs. Age, race, clinical stage, and HER2 status were all significantly associated with biopsy type. However, other variables, including TTT, grade, histology type, hormone receptor, surgery type, and treatment sequence, were not significantly associated with biopsy type.

**Table 3:** Fine-Gray competing risk survival model of main effects and interaction

	sHR (95% CI)	*P* value
TTT		
< 60 days	Reference	
≥ 60 days	1.45 (1.24–1.69)	< 0.001
CNB vs. VAB in		
Stage I	0.92 (0.75–1.12)	0.396
Stage II–III	1.29 (1.15–1.45)	< 0.001
Stage II–III vs. I in		
CNB	5.42 (4.70–6.26)	< 0.001
VAB	3.87 (3.24–4.61)	< 0.001
Biopsy type x stage		0.004

*In addition to the variables above, the model was adjusted for normalized IPW

### CNB is associated with an increased BCSM risk in stage II and III cases

**Fig. 3:**
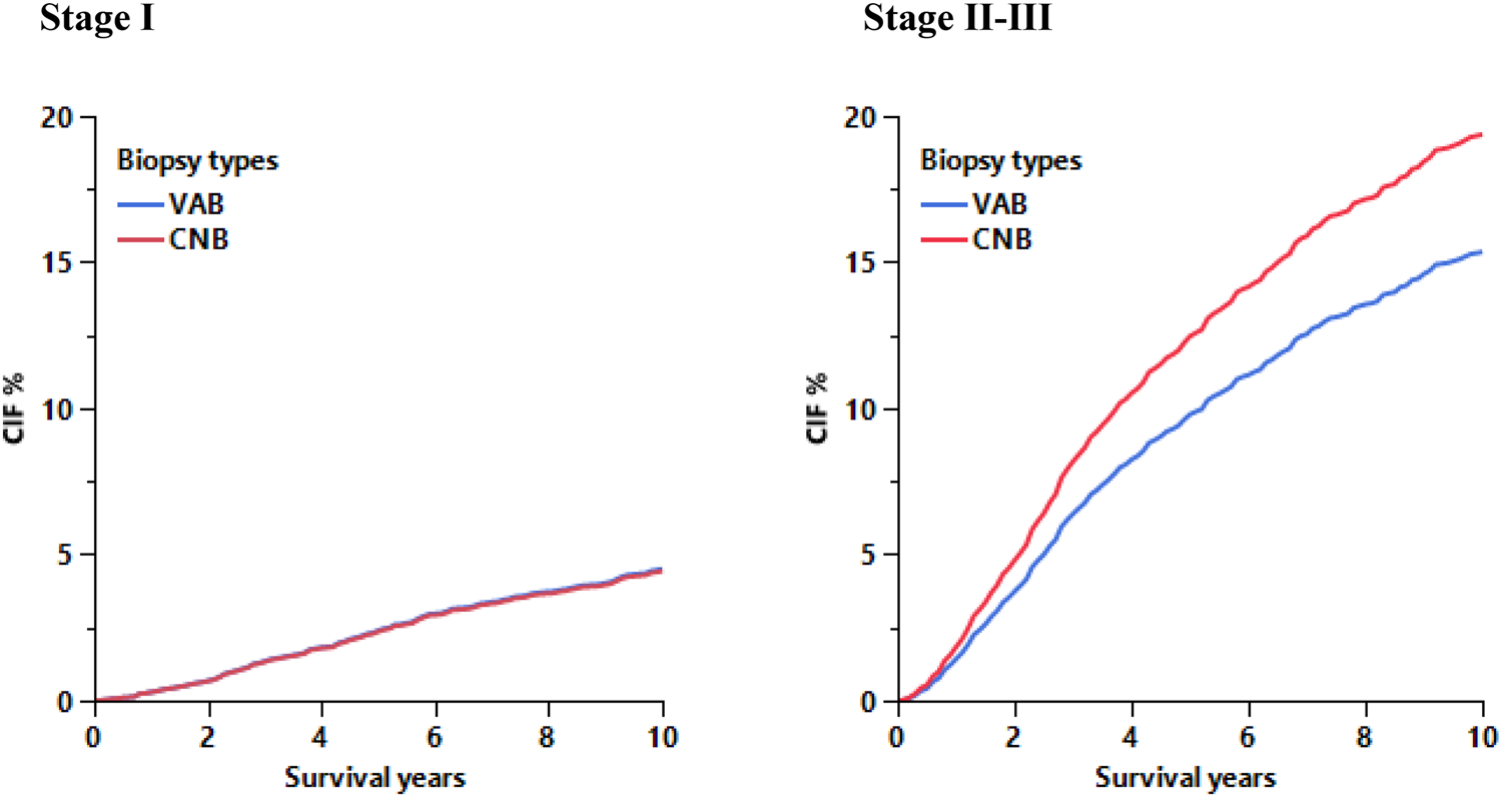
Adjusted cumulative incidence function of breast cancer-specific mortality by biopsy type in stage I and II–III

Further analyses evaluated potential interactions between TTT and biopsy type with other relevant variables. The interaction between TTT and biopsy type was found to be non-significant. Meanwhile, there was a significant interaction between biopsy type and stage (*P* = 0.004; Table 3). CNB was significantly associated with increased risk of BCSM in stage II–III relative to cases who received VAB (sHR: 1.29, 95% CI 1.15–1.45; *P*  < 0.001; Table 3), but there was no significant effect of biopsy type on BCSM in stage I (sHR: 0.92; 95% CI 0.75–1.12; *P* = 0.396; Table 3). BCSM was significantly shorter in Stage II–III as compared to Stage I in CNB group (sHR: 5.42; 95% CI 4.70–6.26; *P* < 0.001) and in VAB group (sHR: 3.87; 95% CI 3.24–4.61; *P* < 0.001; Table 3). Adjusted CIF from this model showed a significantly higher rate of BCSM in stage II–III cases who received CNB compared to VAB, with 2.7% (12.4% vs. 9.7%) higher cumulative incidence at 5 years and 4% (19.4% vs. 15.4%; Fig. 3) at 10 years. In contrast, in stage I patients, the adjusted CIF were indistinguishable between CNB and VAB groups (Fig. 3).

## Discussion

Substantial evidence of increased risk of disease progression and mortality due to treatment delay led the Commission on Cancer to recommend a new quality metric of surgery within 60 days of diagnostic biopsy in stage I–III breast cancer in the non-neoadjuvant setting [19]. Breast cancer is generally considered a slow-growing malignancy [28], as exemplified by the American Cancer Society guideline change from annual to biennial screening mammography for average-risk women 55 years or older based on findings that biennial mammography did not increase advanced stage diagnoses or mortality compared to annual screening [29–34]. Thus, disease progression within 60 days to a level that negatively impacts mortality outcomes appears inconsistent with the nature of this disease, raising the question of whether external factors may promote mortality risk during the period between the diagnostic biopsy and definitive treatment. Currently, no studies provide insight into how increased mortality risk results from treatment delay. Separately, studies have suggested that using CNB for diagnosis is associated with an elevated risk of lymph-node metastasis and distant metastasis [23, 24]. However, a causal relationship between the biopsy type used for diagnosis and treatment delay in breast cancer mortality has never been investigated. Therefore, we explored whether the two most commonly used diagnostic needle biopsy methods (CNB or VAB) differentially influence the mortality risk posed by treatment delay. Our data demonstrated that both TTT ≥ 60 days and diagnosis by CNB were independently associated with a significant increase in adjusted BCSM risk; however, the type of biopsy did not contribute to the increased risk of mortality associated with TTT ≥ 60 days after the biopsy. Further investigation is warranted to identify the root of increased mortality posed by treatment delays.

Numerous biological changes may occur during the period between diagnosis and surgery. In the context of diagnosis, needle biopsy is the first and foremost external force that disrupts tissue integrity. Fourteen-gauge tru-cut^®^ core needle biopsy has been found to leave a persistent needle tract in the remaining tumor until surgical resection [35]. Accordingly, histology pertinent to wound healing is a common change noted around the needle tract of tumors that have undergone needle biopsy [36–39]. We have previously reported that over 70% (*n* = 72) of needle biopsy-proven, surgically resected stage I–II breast tumors display a disproportionate prevalence of macrophages adjacent to the needle tract compared to areas distant from it [40]. Although the cohort size was small, the prevalence of macrophages around the needle tract was sustained regardless of time after biopsy and needle size. A similar line of evidence reported by Weber et al. found an increased level of M2 macrophages in surgically resected biopsied tumors compared to their matched biopsy samples in oral squamous cell carcinomas [41]. Since tumor-associated macrophages (TAMs) with M2 phenotype are noted as a critical factor contributing to disease progression and poorer prognosis in solid tumors, including breast [42], further details to determine the effect of microenvironment changes on treatment delay-associated disease progression and mortality risk are warranted.

Similar to others [23, 24], we found different mortality risk associated with biopsy type, with higher adjusted risk of BCSM in stage II-III patients diagnosed by CNB compared to VAB. There are notable differences in the mode of sampling between CNB and VAB [22]. Both CNB and VAB utilize various needle sizes, ranging from as small as 14-gauge (2 mm) to 8-gauge (4.1 mm) [20]. CNB is typically performed with a high-speed, spring-loaded biopsy device that yields a maximum velocity ranging from 8 to 21 m/s [43] and often requires multiple needle insertions, particularly for large lesions [20, 21]. VAB draws adequate amounts of tissue through typically single insertion of a large-size needle using vacuum-created pressure differentials [44]. Since surrounding tissues absorb energy from the point of the needle [45], larger areas may be affected when multiple insertions of CNB are required [20]. While the clinical significance of cancer cell seeding remains uncertain [46], Diaz et al. identified cancer cell displacement in 37% of specimens obtained with CNB and 23% of VAB (*n* = 352), although the incidence and amount of displacement were inversely related to the interval between biopsy and surgery [47]. Moreover, emerging evidence suggests that cancer cells are capable of sensing mechanical stimuli that are transduced into intracellular signals to modulate migratory behavior [48], essential for metastasis. Further investigation is encouraged to address higher mortality risk in cases diagnosed by CNB.

This study identified two independent modifiable risk factors for BCSM—TTT and CNB. One key strength of this study was the use of Fine-Gray competing risk models, adjusted for IPW for the assessment of BCSM adjusted for clinical and demographic information, as well as the assessment of potential interactions. While this study provides novel insight into the relationship between biopsy type and survival outcomes, there are limitations inherent to the nature of billing claims’ data in the SEER-Medicare database. We minimized potential bias by adjusting our models using IPW for demographic and clinical variables; however, the data do not fully accommodate investigation of the factors underlying treatment delays or the selection of a particular biopsy method, which might vary by hospital and clinical presentation of the disease. In particular, needle gauge, the number of insertions, and specialty of the physician performing the biopsy were unavailable. Additionally, due to changes in the HCPCS code for biopsy procedures, CNB and VAB are no longer distinguishable in cases diagnosed in 2014 or later. Likewise, tumor size, which may affect clinicians’ decision for biopsy type, was unavailable in the database for patients diagnosed after 2004. Although our models were adjusted by group stage, we were unable to negate the possibility that tumor size could be a potential confounder. A similar limitation applied to limited data collection for HER2 status, which became available from 2010 onward. Thus, further examination of the effect in more recent years of diagnosis with more detailed diagnostic information is needed. Before exclusions in our dataset, 7.7% of cases had multiple biopsy dates or types of biopsy performed. While such deviations in biopsy utilization patterns may have an alternative or additive effect on outcomes, such factors were excluded from this study. Furthermore, this study was conducted in a cohort of elderly women with Medicare coverage and should be validated in younger and more diverse populations. Further investigation is necessary to identify the root of increased mortality risk posed by delayed treatment and to explain mortality differences by biopsy type.

## Conclusion

Our study of the SEER-Medicare database showed that TTT ≥ 60 days was an independent predictor of BCSM. Additionally, the use of CNB for diagnosis was significantly associated with an increased likelihood of BCSM compared to those with VAB in stage II–III patients. However, differing types of biopsy did not underlie the increased mortality risk due to treatment delay.

## Supplementary Information

Below is the link to the electronic supplementary material.

Supplementary file1 (PPTX 79 KB)

Supplementary file2 (docx 19 KB)

## Data Availability

The SEER-Medicare data are available to investigators for research purposes on request made to National Cancer Institute and not publicly available due to the agreement.

## Abbreviations

BCSM: Breast cancer-specific mortality
TTT: Time to treatment, i.e., days from diagnosis to the initiation of definitive treatment
CNB: Core needle biopsy
VAB: Vacuum-assisted biopsy
CIF: Cumulative incidence function
sHR: Subdistribution hazard ratio
CI: Confidence interval
AJCC: American joint committee on cancer
ICD-O-3: International classification of disease for oncology, 3rd edition
ICD-9: International classification of disease, ninth revision
NCCN: National comprehensive cancer network
