# Supplementary material for: Increased breast cancer mortality due to treatment delay and needle biopsy type: a retrospective analysis of SEER-medicare
Source: Breast Cancer. 2023 May 3;30(4):627–36. doi: 10.1007/s12282-023-01456-3 (PMC10284985; doi:10.1007/s12282-023-01456-3)
Supplement: Supplementary file 1 — Supplementary file1 (PPTX 79 KB) [file 12282_2023_1456_MOESM1_ESM.pptx]

## Slide 1
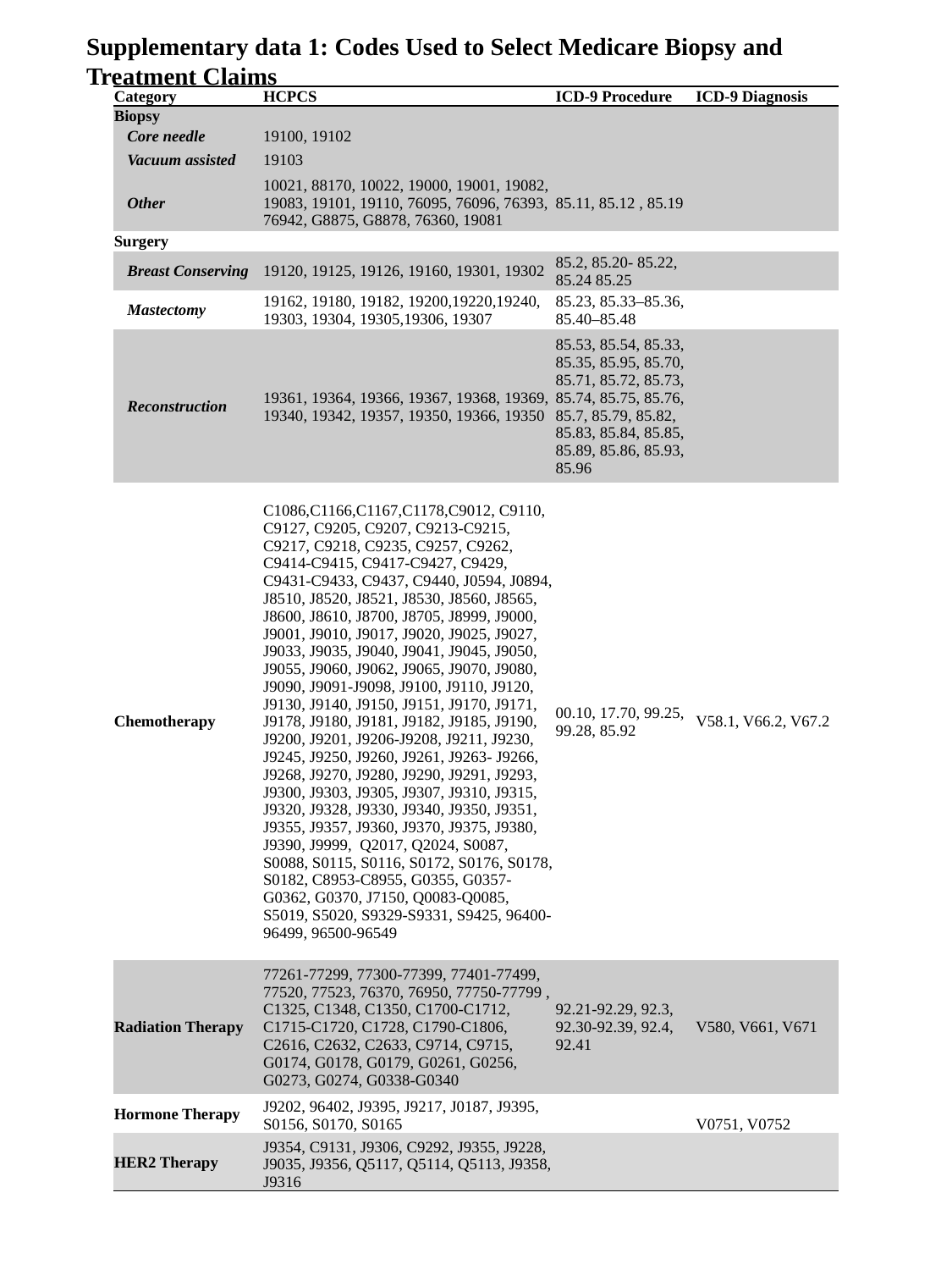

Supplementary data 1: Codes Used to Select Medicare Biopsy and Treatment Claims
| Category | HCPCS | ICD-9 Procedure | ICD-9 Diagnosis |
| --- | --- | --- | --- |
| Biopsy | | | |
| Core needle | 19100, 19102 | | |
| Vacuum assisted | 19103 | | |
| Other | 10021, 88170, 10022, 19000, 19001, 19082, 19083, 19101, 19110, 76095, 76096, 76393, 76942, G8875, G8878, 76360, 19081 | 85.11, 85.12 , 85.19 | |
| Surgery | | | |
| Breast Conserving | 19120, 19125, 19126, 19160, 19301, 19302 | 85.2, 85.20- 85.22, 85.24 85.25 | |
| Mastectomy | 19162, 19180, 19182, 19200,19220,19240, 19303, 19304, 19305,19306, 19307 | 85.23, 85.33–85.36, 85.40–85.48 | |
| Reconstruction | 19361, 19364, 19366, 19367, 19368, 19369, 19340, 19342, 19357, 19350, 19366, 19350 | 85.53, 85.54, 85.33, 85.35, 85.95, 85.70, 85.71, 85.72, 85.73, 85.74, 85.75, 85.76, 85.7, 85.79, 85.82, 85.83, 85.84, 85.85, 85.89, 85.86, 85.93, 85.96 | |
| Chemotherapy | C1086,C1166,C1167,C1178,C9012, C9110, C9127, C9205, C9207, C9213-C9215, C9217, C9218, C9235, C9257, C9262, C9414-C9415, C9417-C9427, C9429, C9431-C9433, C9437, C9440, J0594, J0894, J8510, J8520, J8521, J8530, J8560, J8565, J8600, J8610, J8700, J8705, J8999, J9000, J9001, J9010, J9017, J9020, J9025, J9027, J9033, J9035, J9040, J9041, J9045, J9050, J9055, J9060, J9062, J9065, J9070, J9080, J9090, J9091-J9098, J9100, J9110, J9120, J9130, J9140, J9150, J9151, J9170, J9171, J9178, J9180, J9181, J9182, J9185, J9190, J9200, J9201, J9206-J9208, J9211, J9230, J9245, J9250, J9260, J9261, J9263- J9266, J9268, J9270, J9280, J9290, J9291, J9293, J9300, J9303, J9305, J9307, J9310, J9315, J9320, J9328, J9330, J9340, J9350, J9351, J9355, J9357, J9360, J9370, J9375, J9380, J9390, J9999,  Q2017, Q2024, S0087, S0088, S0115, S0116, S0172, S0176, S0178, S0182, C8953-C8955, G0355, G0357-G0362, G0370, J7150, Q0083-Q0085, S5019, S5020, S9329-S9331, S9425, 96400-96499, 96500-96549 | 00.10, 17.70, 99.25, 99.28, 85.92 | V58.1, V66.2, V67.2 |
| Radiation Therapy | 77261-77299, 77300-77399, 77401-77499, 77520, 77523, 76370, 76950, 77750-77799 , C1325, C1348, C1350, C1700-C1712, C1715-C1720, C1728, C1790-C1806, C2616, C2632, C2633, C9714, C9715, G0174, G0178, G0179, G0261, G0256, G0273, G0274, G0338-G0340 | 92.21-92.29, 92.3, 92.30-92.39, 92.4, 92.41 | V580, V661, V671 |
| Hormone Therapy | J9202, 96402, J9395, J9217, J0187, J9395, S0156, S0170, S0165 | | V0751, V0752 |
| HER2 Therapy | J9354, C9131, J9306, C9292, J9355, J9228, J9035, J9356, Q5117, Q5114, Q5113, J9358, J9316 | | |

## Slide 2
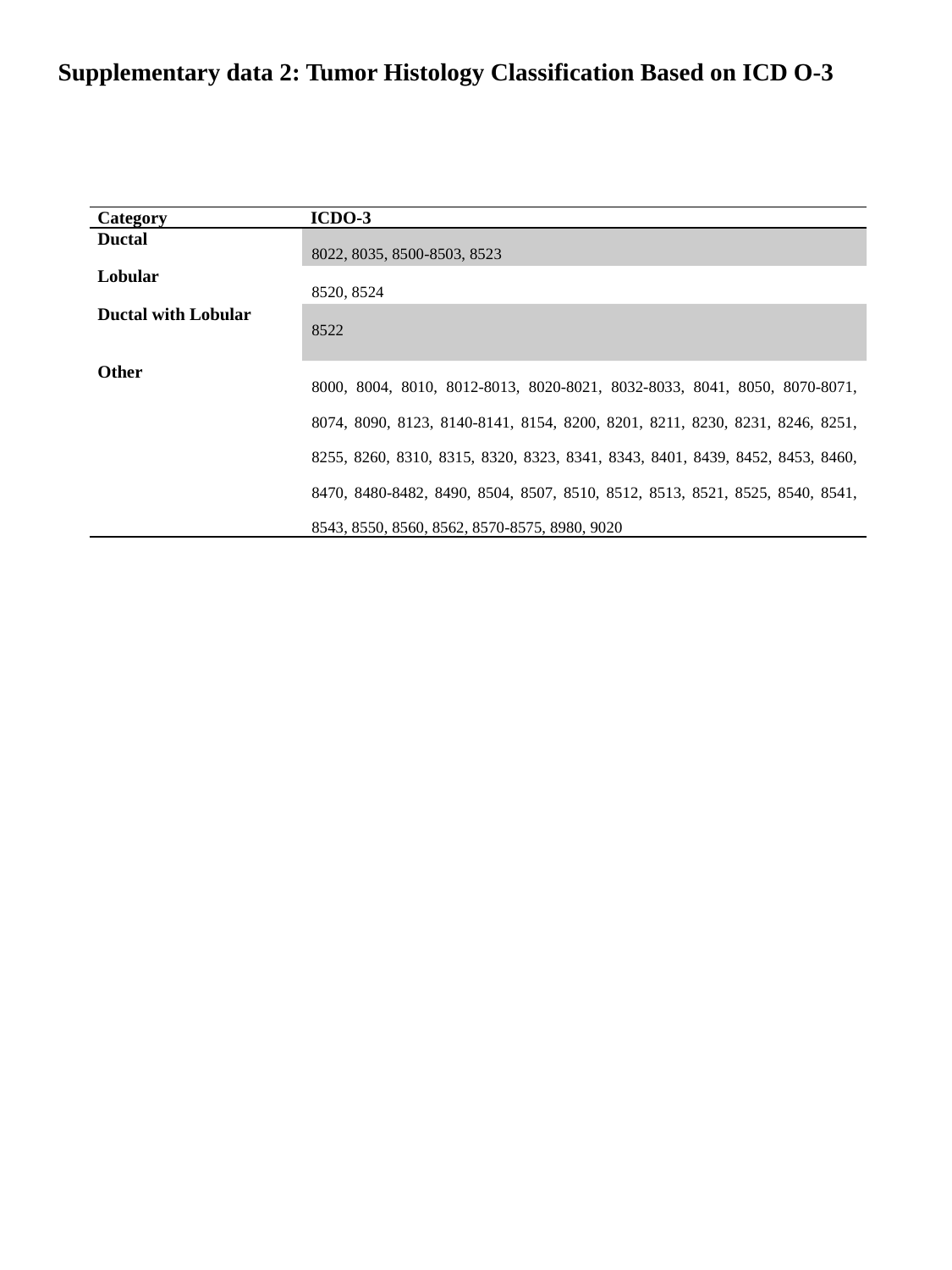

Supplementary data 2: Tumor Histology Classification Based on ICD O-3
| Category | ICDO-3 |
| --- | --- |
| Ductal | 8022, 8035, 8500-8503, 8523 |
| Lobular | 8520, 8524 |
| Ductal with Lobular | 8522 |
| Other | 8000, 8004, 8010, 8012-8013, 8020-8021, 8032-8033, 8041, 8050, 8070-8071, 8074, 8090, 8123, 8140-8141, 8154, 8200, 8201, 8211, 8230, 8231, 8246, 8251, 8255, 8260, 8310, 8315, 8320, 8323, 8341, 8343, 8401, 8439, 8452, 8453, 8460, 8470, 8480-8482, 8490, 8504, 8507, 8510, 8512, 8513, 8521, 8525, 8540, 8541, 8543, 8550, 8560, 8562, 8570-8575, 8980, 9020 |

## Slide 3
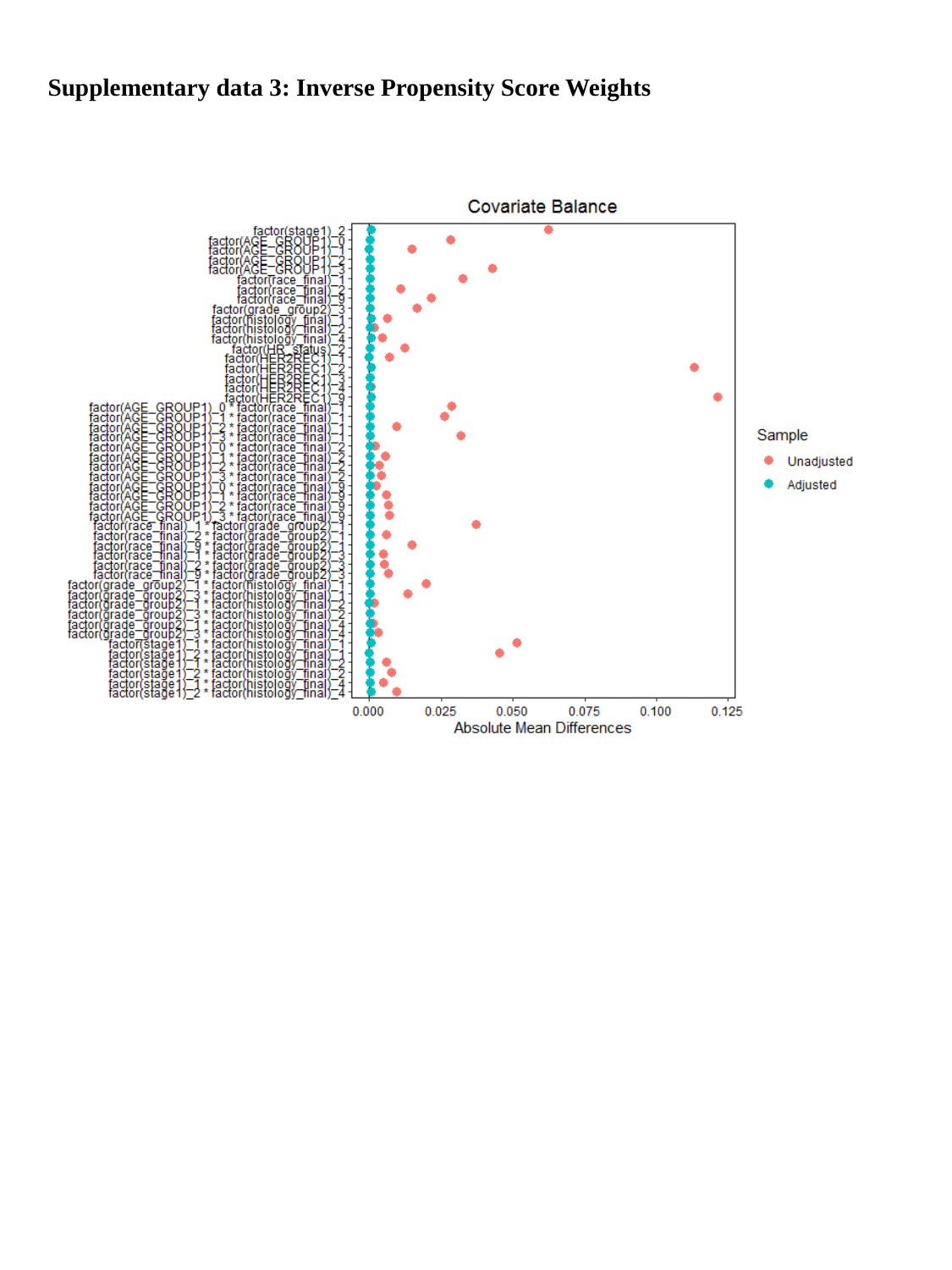

Supplementary data 3: Inverse Propensity Score Weights
