# Supplementary material for: Increased breast cancer mortality due to treatment delay and needle biopsy type: a retrospective analysis of SEER-medicare
Source: Breast Cancer. 2023 May 3;30(4):627–36. doi: 10.1007/s12282-023-01456-3 (PMC10284985; doi:10.1007/s12282-023-01456-3)
Supplement: Supplementary file 2 — Supplementary file2 (docx 19 KB) [file 12282_2023_1456_MOESM2_ESM.docx]

**Supplementary Data 4. Results of logistic regression model for biopsy type assignment (propensity model)**

|  | **Odds Ratio (95% CI)** | ***P* value** |
| --- | --- | --- |
| **TTT** |  | 0.914 |
| <60 days vs. ≥60 days | 1.01 (0.92-1.10) |  |
| **Age** |  | <.0001 |
| ≤69 vs. >80 | 0.76 (0.72-0.82) |  |
| 70-75 vs. >80 | 0.82 (0.77-0.87) |  |
| 75-80 vs. >80 | 0.86 (0.81-0.92) |  |
| **Race** |  | <.0001 |
| White vs. Other | 0.66 (0.59-0.72) |  |
| Black vs. Other | 0.80 (0.70-0.91) |  |
| **Grade** |  | 0.947 |
| 1-2 vs. 3 | 1.00 (0.94-1.06) |  |
| **Stage** |  | <.0001 |
| I vs. II-III | 0.78 (0.74-0.82) |  |
| **Histology** |  | 0.574 |
| Ductal vs. Other | 0.95 (0.87-1.04) |  |
| Lobular vs. Other | 0.95 (0.86-1.05) |  |
| **Hormone Receptor** |  | 0.525 |
| Positive vs. Negative | 0.98 (0.91-1.05) |  |
| **Type of Surgery** |  | 0.091 |
| Breast Conserving vs. Mastectomy with reconstruction | 1.16 (1.01-1.34) |  |
| Mastectomy vs. Mastectomy with reconstruction | 1.17 (1.02-1.36) |  |
| **Treatment Sequence** |  | 0.466 |
| Neoadjuvant vs. Surgery First | 1.05 (0.92-1.21) |  |
| **HER2** |  | <.0001 |
| Positive vs. Unknown | 0.64 (0.57-0.72) |  |
| Negative vs. Unknown | 0.60 (0.57-0.63) |  |
| Borderline vs. Unknown | 0.77 (0.61-0.96) |  |
